# Rethinking knee injury prevention strategies: joint-by-joint training approach paradigm versus traditional focused knee strengthening

**DOI:** 10.5114/biolsport.2025.148544

**Published:** 2025-04-14

**Authors:** Wissem Dhahbi, Olivier Materne, Karim Chamari

**Affiliations:** 1 Research Unit “Sport Sciences, Health and Movement”, High Institute of Sports and Physical Education of Kef, University of Jendouba, Kef, Tunisia; 2 Qatar Police Academy, Police College, Training Department, Doha, Qatar; 3 Sports Medicine Department, Standard de Liege Football Club, Belgium; 4 Research Department, Naufar, Wellness and Recovery Centre, Doha, Qatar; 5 High Institute of Sport and Physical Education of Ksar Said, University of Manouba, Manouba, Tunisia

**Keywords:** Biomechanical adaptation, Functional movement screening, Locomotion, Injury biomechanics, Kinetic chain dynamics, Motor skills, Postural control, Proprioception

## Abstract

Knee injuries remain a significant challenge in competitive sports, impacting athletic performance, long-term joint health, and healthcare resource utilization. Traditional prevention strategies often focused primarily on strengthening the knee itself. However, emerging evidence supports a joint-by-joint training approach (JBJTA), emphasizing the interconnected kinetic chain, particularly the trunk, hips, ankles, and feet. This commentary explores the potential of JBJTA in knee injury prevention by enhancing hip and ankle mobility, trunk and foot stability, and proprioception. The JBJTA posits that optimal joint function and adaptability across the kinetic chain contribute to reduced knee stress and improved dynamic stability. Limited hip range of motion (ROM) and ankle dorsiflexion are associated with altered mechanics, such as increased knee valgus, which increases the risk of anterior cruciate ligament (ACL) injuries. Additionally, foot stability and proprioception are crucial for dynamic balance and neuromuscular control, further mitigating knee injury risk. Furthermore, we highlight the importance of the JBJTA for biomechanical knee load redistribution, optimizing force distribution throughout the lower limb to alleviate knee stress. The approach enhances lower limb alignment, shock absorption, and efficient force transfer, contributing to reduced knee valgus and overall injury risk. The JBJTA offers a comprehensive strategy for knee injury prevention by addressing the interdependence of the kinetic chain. This paradigm shift from isolated knee strengthening to a holistic approach promises improved performance and long-term musculoskeletal health for athletes. We call for future research to rigorously evaluate the efficacy of this approach in diverse athletic populations.

## INTRODUCTION

Knee injuries impose a substantial burden on athletes across all sports, with significantly higher rates observed in female compared to male athletes [1, 2]. These injuries impact both immediate athletic performance and long-term joint health, while placing substantial demands on healthcare resources [3]. Traditional knee-injury prevention focuses on the knee itself [4, 5]. However, recent biomechanical analyses demonstrate emerging evidence suggesting that a more comprehensive approach targeting the interconnected kinetic chain, particularly the trunk, hips and ankles, could result in more efficient outcomes compared to a knee-focused approach [6–8].

The incidence and biomechanical mechanisms of knee injuries demonstrate significant sex-specific differences, particularly in jumping and cutting sports [9]. In the context of the latter sports, it has been observed that women exhibit a 2–4 times greater risk of anterior cruciate ligament (ACL) injury compared to men [10]. Multiple factors contribute to this disparity, including differences in neuromuscular control, particularly for the trunk and hip [2], landing mechanics [11], and anatomical alignment. Young athletes, especially those in the rapid stages of development, encounter unique challenges [12]. The development of musculoskeletal systems and the presence of neuromuscular disorders elevate the risk of injury [13]. These sex-specific differences in injury mechanisms necessitate targeted prevention strategies that address the unique biomechanical risk factors present in female athletes [14–16].

We explore the potential of a joint-by-joint training approach (JBJTA) to enhance knee injury prevention strategies. Kinematic analyses of injury mechanisms and biomechanical studies demonstrate the critical role of hip and ankle mobility, alongside trunk and foot stability, in reducing knee stress. These findings emphasize the need to address the interconnectedness of these areas to improve dynamic stability and movement control. JBJTA aligns with this understanding by focusing on optimizing movement patterns across multiple joints, thereby reducing knee loading during high-risk athletic activities. This evidence-based approach provides a framework for developing more effective injury prevention programs.

## A holistic paradigm for injury prevention and postural adaptation

The JBJTA addresses the interdependent relationship between joints, where trunk control and lower extremity alignment work synergistically during dynamic movement patterns [17–19]. Disruptions in this interplay lead to compensatory movements and increase the risk of knee injury [20]. The JBJTA emphasizes interconnectedness within the kinetic chain, recognizing that optimal movement at one joint will influence the cranial and caudal joint movement pattern [19, 21, 22].

This approach shifts focus from static posture correction to dynamic postural change response [23, 24]. Enhanced neuromuscular control across the kinetic chain, particularly of the trunk and hip, is associated with reduced knee loading patterns during high-risk athletic maneuvers [17, 18, 20]. By acknowledging the body’s natural ability to adapt, the JBJTA promotes a more sustainable and effective strategy for knee injury prevention [4, 7, 25].

Recent biomechanical analyses demonstrate that differences in trunk control among female athletes are associated with increased knee abduction moments and altered landing mechanics [1]. During landing tasks, increased lateral trunk motion is associated with higher ground reaction forces and knee valgus angles for the ipsilateral leg [11]. These findings highlight the importance of addressing trunk control within injury prevention programs.

## Biomechanical principles of lower-limb joint functionality

### Trunk Control

Trunk neuromuscular control significantly influences knee injury risk through modulation of lower extremity biomechanics and load distribution. Recent prospective studies have demonstrated that decreased trunk control predicts future knee injury risk [1, 26]. A thorough examination conducted by Sabet et al. [26] established a correlation between diminished trunk control and increased knee valgus angles and moments during dynamic exercises, thereby identifying variables associated with the risk of ACL injury. Proprioceptive repositioning errors demonstrated a 2.5-fold increased risk of knee injury among athletes exhibiting inadequate trunk control [27]. During landing exercises, trunk stability enhances lower extremity kinematics and diminishes ground reaction forces [28]. The influence of the trunk extends beyond local stability. Song et al. [2] identified a higher frequency of knee abduction events and lateral trunk tilt during cutting movements, which may contribute to an increased risk of ACL injuries. Effective management of trunk motion across multiple planes is therefore essential for the development of preventative programs.

## Hip Mobility

Restricted hip mobility, particularly in rotation and extension, alters lower extremity kinetic chain mechanics during dynamic tasks and is associated with an increased risk of knee injuries [29–33]. Studies by Bathe et al. [31] and Mai et al. [32] link restricted hip mobility to altered mechanics, including increased knee valgus (inward collapse) during dynamic movements – a known risk factor for anterior cruciate ligament (ACL) tears. Furthermore, targeted hip mobility exercises have been shown to improve landing mechanics and reduce knee valgus moments [21, 25]. This suggests that adequate hip mobility is crucial for proper knee function and injury prevention, especially in athletes performing activities with high risk of non-contact knee injuries [5, 34, 35].

## Ankle Mobility

Limited ankle dorsiflexion ROM alters landing mechanics and increases knee valgus angles during dynamic tasks [36–38]. Studies by Zamankhanpour et al. [37] and Hewett et al. [38] link restricted dorsiflexion to altered landing mechanics, including increased knee valgus (inward collapse) – a known risk factor for ACL tears [39]. Conversely, targeted ankle mobility exercises can improve landing mechanics and reduce ACL injury risk [40]. Hamoongard et al. [40] showed an 8-week program enhanced dorsiflexion ROM and decreased peak knee abduction moments during cutting maneuvers. Similarly, Bell et al. [41] reported an improvement by 20% to 50% in knee valgus angles following ankle mobility training. These findings suggest that maintaining adequate ankle dorsiflexion ROM is crucial for proper knee function and contributes to injury prevention [36–38, 41].

## Foot Stability and Proprioception: The Sensory Guardian of the Knee

Foot stability and neuromuscular control represent crucial yet often overlooked components of the kinetic chain that influence knee loading patterns to reduce the knee injury risk [42, 43]. Indeed, deficits in these areas disrupt the kinetic chain, leading to improper load distribution and altered gait patterns that increase the risk of knee valgus (inward collapse), a primary risk factor for knee injuries [42]. Combined foot strengthening and proprioceptive training has been shown to improve dynamic balance, reduce knee valgus angles during landings [44, 45], and enhance neuromuscular control throughout the lower limb. This leads to reduced resultant intersegmental loads between the shank and thigh, as computed via inverse dynamics [46]. Proprioceptive training of the foot and ankle complex can also significantly improve dynamic postural control and knee joint stability [44]. Studies further suggest that incorporating these exercises into training programs can significantly reduce the incidence of knee injuries [42, 44]. By promoting foot stability and proprioception, the effectiveness of injury prevention programs could be enhanced [40, 43].

## Biomechanical knee load redistribution

Recent research suggests a multi-joint approach for optimizing force distribution throughout the trunk and lower limb, potentially reducing knee stress [43, 47, 48]. Studies by King et al. [49] using 3D motion capture demonstrate that improved hip and ankle mobility, along with enhanced foot proprioception, led to a more even distribution of joint moments during cutting maneuvers [47]. Notably, this redistribution was associated with a significant reduction in peak knee valgus, a known risk factor for ACL injuries [47, 49].

The enhancement of hip and ankle mobility, combined with proper trunk control, creates more efficient load distribution patterns through the lower extremity kinetic chain [27]. This improved biomechanical efficiency results in reduced peak knee joint loads during high-risk athletic maneuvers. This likely stems from improved lower limb alignment, enhanced shock absorption at the ankle, and a more efficient transfer of forces across the entire extremity [43, 50]. The effectiveness of incorporating strength, balance and movement control exercises in injury prevention programs, particularly for reducing knee and ACL injuries, is well-supported by numerous studies [47, 49]. This evidence emphasizes the importance of targeting not only the knee itself, but also the surrounding joints that significantly influence the lower-limb biomechanics.

## Mechanisms for knee injury prevention: a multi-by-joint approach

**FIG. 1:**
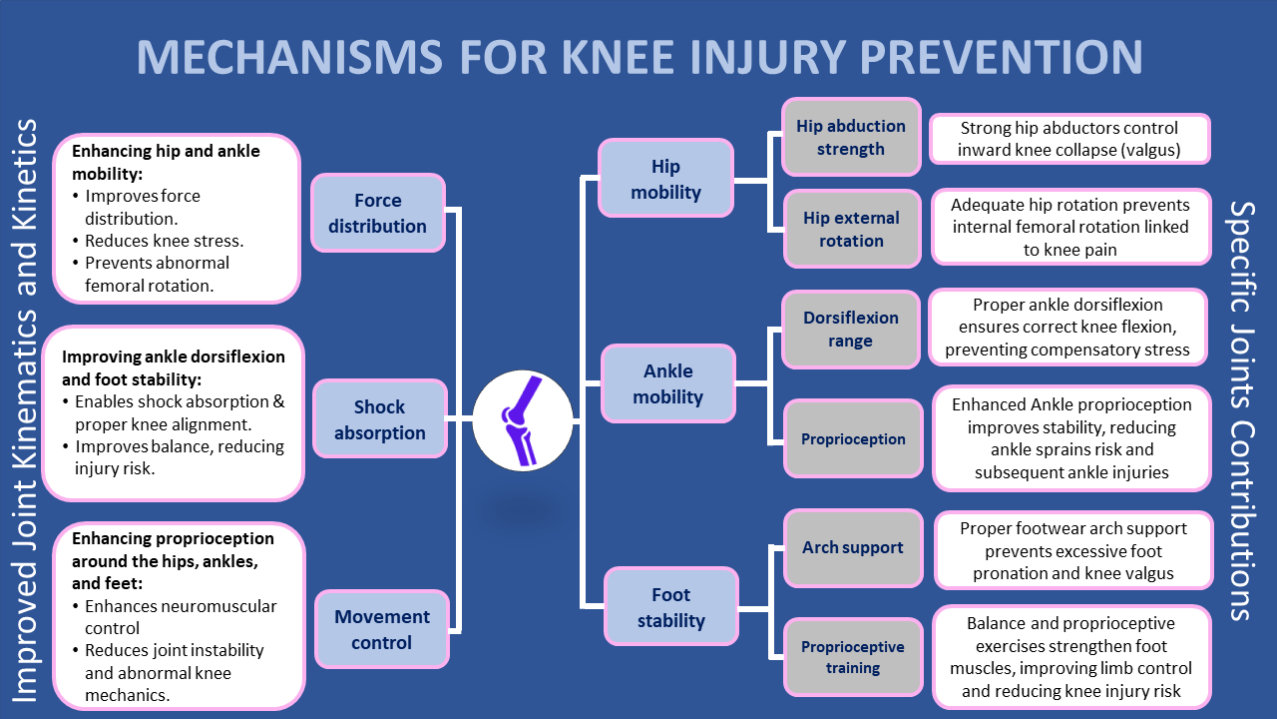
Mechanisms for knee injury prevention based on joint-by-joint training approach.

Current evidence demonstrates that optimizing biomechanics throughout the lower extremity requires addressing four key mechanisms [1, 2, 6]. These mechanisms have been validated through biomechanical analyses and prospective studies that demonstrate their effectiveness in reducing knee injury risk. The validation methods include 3D motion capture, electromyography (EMG) analysis, and force plate measurements during dynamic tasks [51, 52]. This approach focuses on four key mechanisms (Figure 1):

**– Improved Joint Kinematics and Kinetics:** Enhanced hip and ankle mobility promotes efficient force distribution, reducing peak loads on the knee [30, 38]. Improved ankle dorsiflexion and foot stability enhance shock absorption [39, 45], mitigating impact forces [37, 46].
**– Specific Joint Contributions:** Strengthening key muscles within the kinetic chain addresses individual movement deficiencies [1, 48]. For instance, strong hip abductors like the gluteus medius control valgus, while sufficient ankle dorsiflexion ROM allows proper knee flexion during activities like squatting [39, 48]. Additionally, ankle proprioception and proper arch support contribute to dynamic stability and alignment, reducing the risk of injuries that can indirectly affect the knee [39].
**– Enhanced Lower Limb Control:** Balance exercises and proprioceptive drills specifically target the foot muscles, further improving lower limb control and stability, offering an additional layer of protection against knee injuries [38]. Furthermore, better proprioception around the hips, ankles, and feet improves neuromuscular control, minimizing abnormal mechanics at the knee [42].
**– Trunk Control:** Enhanced trunk neuromuscular control and core stability contribute to a reduction in dynamic knee valgus angles and moments [26]. Trunk positioning and movement patterns contribute to the reduction of peak loads on the knee by facilitating an even distribution of pressures throughout the lower extremities [2]. Exercises specifically designed for trunk-based prevention contribute to the alignment of the lower limbs, the management of ground reaction forces, and the mechanics of landing [26, 28].
**– Non-Linear Dynamic Exercise Approach**: Non-linear dynamic exercise programs more accurately represent the volatility inherent in sports compared to linear programs. The alteration of weights, velocities, and trajectories in exercise regimens serves to evaluate the neuromuscular system’s capacity to adapt to unforeseen variations [53]. Exercises conducted on unstable surfaces and perturbation training have the potential to enhance joint proprioception and dynamic stability [54]. Gokeler et al. [55] demonstrated that non-linear exercises enhance landing mechanics and reduce knee valgus moments within ACL injury prevention programs for female athletes. This approach enhances the cognitive-motor abilities essential for athletic performance and contributes to the mitigation of physical injuries [56].

By addressing these mechanisms, this multi-joint approach will potentially optimize movement patterns by distributing forces more effectively across the lower extremity and consequently improving the knee joint resilience, ultimately leading to a more robust and stable knee joint (Figure 1).

## Clinical implications and future directions

### Validation Methodology

The efficacy of the JBJTA requires comprehensive validation across multiple assessment domains. Biomechanical assessment forms the foundation, encompassing three-dimensional motion capture during sport-specific movements to analyze movement patterns, ground reaction force analysis during landing tasks to evaluate loading characteristics, and electromyographic analysis of trunk and lower extremity muscles to understand neuromuscular activation patterns. These technical measurements are complemented by clinical measures including systematic range of motion measurements for hip and ankle joints, comprehensive balance and proprioception testing, and functional movement screening to assess movement quality and identify potential compensatory patterns. Performance metrics provide quantifiable indicators of movement quality and neuromuscular control, including vertical jump height, multi-planar landing mechanics, change-of-direction efficiency, and dynamic postural stability measures. These metrics offer objective data on the training approach’s impact on athletic performance capabilities. The validation process is further enhanced by systematic injury surveillance, which involves prospective tracking of injury rates across participating athletes, detailed analysis of injury mechanisms when they occur, and monitoring of return-to-sport success rates following rehabilitation. This multifaceted validation approach ensures a thorough evaluation of the JBJTA’s effectiveness in both preventing injuries and optimizing athletic movement performance.

## Implementation Guidelines and Practical Recommendations

Implementation of JBJTA requires systematic baseline screening and ongoing monitoring. The initial assessment should include a functional movement screen, dynamic trunk control evaluation, detailed hip and ankle mobility measurements, and single-leg landing analysis. Progress monitoring utilizes movement quality scoring, specific load progression criteria, and performance metrics tracking. Sports medicine practitioners should incorporate these validated assessment tools alongside targeted interventions for hip and ankle mobility, trunk and foot stability, and proprioception:

**– Comprehensive Corrective Exercise Programs (CCEPs):** CCEPs should be implemented to address hip, ankle, and foot function through an integrated combination of strength training, neuromuscular control drills, and proprioception exercises. To ensure optimal outcomes, these programs require systematic monitoring using multiple assessment methods. Three-dimensional motion analysis provides detailed insights into joint kinematics during dynamic tasks, while kinetic measurements enable precise evaluation of ground reaction forces and loading patterns throughout movement sequences. Muscle activation patterns can be quantified through EMG analysis, offering valuable data about neuromuscular coordination and timing.
  Progress should be tracked using Functional Movement Screening (FMS) tools to assess movement quality and identify areas needing attention. Regular goniometric measurements of ROM help monitor joint mobility improvements, while dynamic stability can be evaluated through standardized balance assessments such as the Y-Balance Test and Single Leg Hop Test [57, 58]. This comprehensive monitoring approach ensures that interventions can be adjusted based on objective measures of progress and performance.
**– Individualized Assessment:** Conduct systematic biomechanical screening to identify movement pattern deficiencies, considering individual variations in trunk control, hip function, and landing mechanics.
**– Sport-Specific Considerations:** Adapt prevention strategies based on sport-specific biomechanical demands and injury mechanisms identified through kinematic analyses and laboratory studies.

Implementation follows four progressive phases: (1) movement pattern assessment using validated screening tools, (2) fundamental movement competency development focusing on quality and control, (3) systematic progression of movement complexity and load intensity, and (4) sport-specific skill integration under varying temporal and spatial constraints. Advancing through phases requires meeting quantifiable criteria based on movement quality and neuromuscular control measures. This structured approach ensures appropriate progression while maintaining a focus on optimal movement patterns [2, 11].

Future research should focus on long-term prospective studies to evaluate the efficacy of this multi-joint approach in reducing knee injury burden across diverse sports populations.

## CONCLUSIONS

Biomechanical analyses and prospective studies demonstrate that non-contact knee injuries result from complex interactions between trunk control, hip function, and lower extremity alignment. Traditional approaches focusing solely on isolated knee strengthening often fail to adequately address these comprehensive risk factors.

The JBJTA addresses fundamental biomechanical causes of excessive knee stress by optimizing movement patterns throughout the kinetic chain. Enhanced mobility of the hips and ankles, combined with improved trunk control and foot stability, promotes more efficient force distribution patterns during athletic tasks.

This evidence-based approach would enable athletes to develop more efficient movement strategies while minimizing injury risk. Implementation of comprehensive prevention programs based on these principles should consider individual movement patterns, sport-specific demands, and identified risk factors.
